# S-1 plus docetaxel: a safe and effective chemotherapy regimen for stage III gastric cancer

**DOI:** 10.1186/s40880-019-0408-2

**Published:** 2019-10-26

**Authors:** Feiyu Diao, Wei Lai, Zhonghua Chu

**Affiliations:** 1 Department of General Surgery, Sun Yat-Sen Memorial Hospital, Sun Yat-Sen University, Guangzhou, Guangdong P. R. China; 2 Guangdong Provincial Key Laboratory of Malignant Tumor Epigenetics and Gene Regulation, Department of Gastrointestinal Surgery, Sun Yat-sen Memorial Hospital, Sun Yat-sen University, Guangzhou, 510120 Guangdong P. R. China

## Main text

Gastric cancer (GC) is the fourth most common cancer worldwide [1], with approximately 50% of GC cases and deaths occurring in China [2]. Gastrectomies have the potential to remove all visible tumor tissues and obtain histologically free-margins, which have shown to provide curative reliability in patients with early-stage GC [3, 4]. However, < 25% of GC cases are diagnosed with early-stage disease. The survival of the remaining patients falls below 50% [5], even though after undergoing D2 gastrectomy or acquired targeted therapy [6, 7]; thereby prompting more commitment in finding other alternatives in the hope of improving the patient outcomes. Results from two landmark trials in 2001 and 2006 have demonstrated that adjuvant therapies using post-operative chemo-radiotherapy and perioperative chemotherapy (CT) were effective therapeutic options [8, 9]. Currently, both of them are accepted standards of care in the West.

Publication of landmark trial in 2007 (the Adjuvant Chemotherapy Trial of S-1 for Gastric Cancer) demonstrated that patients with stage II or III GC receiving 12-month of postoperative S-1 treatment could benefit from a significantly longer overall survival (OS) and relapse-fee survival (RFS) as compared with those had surgical recession alone [10]. Recently, the adjuvant CT trial of capecitabine-oxaliplatin doublet for GC has demonstrated similar survival benefit [11]. Therefore, Chinese GC patients currently have two standard treatments with similar efficacy [12]. However, because of the poor outcome for stage III GC patients who undergo the S-1 monotherapy, better alternatives are being relentless researched [13]. A previous study has shown that docetaxel has efficacy in combination with S-1 [14]. But there is still no evidence regarding the safety and efficacy of S-1-docetaxel doublet in the treatment of stage III GC. In a study recently published in *Journal of Clinical Oncology*, titled “Addition of docetaxel to oral fluoropyrimidine improves efficacy in patients with stage III gastric cancer: interim analysis of JACCRO GC-07, a randomized controlled trial”, Yoshida et al. [15] conducted a randomized phase III study to investigate the superiority of postoperative S-1 plus docetaxel over S-1 alone for R0 resection of pathologic stage III GC.

In that study, the authors planned to enroll 1100 patients from April 2013 to December 2017 at 138 study sites in Japan. They conducted the second interim analysis in April 2017 when the number of events reached 216 among 915 enrolled patients. Of these cases, 341 patients have been treated with S-1 plus docetaxel and 348 treated with S-1 alone. The median follow-up time was 12.5 months. The baseline characteristics were well-balanced between the two groups. From the second interim analysis, the authors found that the 3-year RFS in the S-1 plus docetaxel group was 66% (95% confidence interval [CI] 59%–73%) as compared to 50% (95% CI 41%–58%) in the S-1 group; indicating that the RFS rate of S-1 plus docetaxel group was statistically higher than that of S-1 group (hazard ratio = 0.632; 99.99% CI 0.400–0.998; *P* < 0.001). Therefore, the enrollment was prematurely terminated as recommended by the independent data and the safety monitoring committee. The adverse event analyses, particularly for neutropenia and leukopenia, demonstrated that the incidences of grade 3 or greater adverse events were higher in the S-1 plus docetaxel group as compared to the S-1 group. Fortunately, all adverse events were manageable.

Since the era of comparisons with gastrectomy alone, no critical trials have been conducted to explore the advantages of more intensive postoperative CT over S-1 monotherapy, however, the present study has filled this gap. A previous study found that a combination of S-1 and cisplatin was poorly tolerated in the postoperative adjuvant CT for advanced GC in Japan [16]. Additionally, a phase III clinical trial comparing the efficacy of S-1 combination with docetaxel and S-1 monotherapy showed significant improvement in time to progression of advanced GC patients receiving S-1 plus docetaxel [17]. Furthermore, previous feasibility study has indicated that S-1 combination with docetaxel was well-tolerated after gastrectomy [18]. Thus, the combination of S-1 and docetaxel aroused the attentions of clinicians. In the present study, this regimen met the predetermined hypothesis for more than 15% improvement in 3-year RFS at the second interim analysis and the RFS benefit was accompanied by a favorable safety profile. These findings can be applicable in countries in which perioperative CT or chemoradiation is not standard.

The limitations of this study include: (1) the number of deaths at the time of the interim analysis was relatively small, thus, the OS data should be cautiously reconsidered; (2) the early termination of the study at the interim analysis; (3) The patient number at risk did not reach the desired number, which may further delimit the observed findings; and (4) the survival difference between the two groups may have been a result of the poor 3-year RFS of patients in the S-1 group, which was far lower than the pretrial estimation. Therefore, the tremendous research still needs to be done in the future, including but not limiting to, (1) conducting additional follow-up for future evaluation of secondary endpoints that include OS; (2) recalculate the sites of relapse at such endpoints.

## Data Availability

Not applicable.

## Abbreviations

GC: gastric cancer
CT: chemotherapy
OS: overall survival
RFS: relapse-fee survival
CI: confidence interval
